# Progress in Psychiatry

**Published:** 1900-10-20

**Authors:** 


### Progress in Psychiatry.

(*Continued from page* 32.)

**Toxic Action of Cocaine and other Drugs.**—Cocaine and preparations containing the drug have only recently taken a place among the toxic causes of cerebral degeneration and insanity, although in Peru the habit of chewing coca leaves had been practised by the Indians for centuries. A confirmed chewer of coca, says Johnston,[67] is called a *coquero*, and he becomes more thoroughly a slave to the leaf than the inveterate drunkard is to alcohol. The inveterate *coquero* has an unsteady gait, yellow skin, quivering lips, hesitant speech, and general apathy. Since the introduction of the drug (the coca leaf and its alkaloidal preparations) into Europe and North America its misuse has become a noteworthy vice among many classes of society. This cocaine-habit is undoubtedly growing in extent and seriousness. Many of the cases reported as due to cocaine are, says Dr. Talbot,[68] attributable to the desire of the hysteric or neurasthenic to secure a new sensation. The habit is very frequently induced by patent medicines taken by hysterical and neurasthenic subjects to cure nervousness, fatigue and nerve-exhaustion, or to apply stimulus to a jaded life. A notorious snuff—advertised in newspapers and magazines, for colds and catarrh—is said by Talbot to contain 3 per cent. of cocaine, and is frequently taken by neurotic youths for such ailments, with the result that the nervous trouble is aggravated. Coca wines are sold by grocers and chemists, and are not always taken with discretion by patients convalescing from influenza or other fevers. Hypodermic injections of cocaine are resorted to by the jaded and blasé men and women who live Bohemian lives or are continually suffering from *ennui* and feel the need of novel exhilarants and stimulants.

In the treatment of alcoholic craving and the morphia habit many careful observers pronounce cocaine to be of great benefit.[69] It restores appetite, induces sleep, and promotes digestion, while it soothes the brain and induces a feeling of contentment and calm. On the other hand, many cases are reported of persons who have relinquished the morphia habit only to become addicted to cocaine. While small doses of cocaine increase muscular power and endurance and diminish the sense of fatigue, large doses have a different effect. In animals (dogs) moderate doses cause excitement and exhilaration, with expressions of joy and delight. Larger doses produce dejection, terror, trembling, and even convulsions. In man small doses, e.g., one-fifth of a grain hypodermically injected produce exhilaration and cerebral excitement, but with increase of the dose to $\frac{1}{2}$ or $\frac{3}{4}$ grain there result tachycardia, irregular action of the heart, dyspnœa, loss of self-control, and mental confusion passing into unconsciousness (Hammond). Erlenmeyer in Germany, Magnan in France, and Chalmers da Costa in America, have described and called attention in recent years to the cerebral disorders produced by the abuse of cocaine. In most of the cases observed the intoxication has been a mixed one, viz., with morphia and cocaine simultaneously. The special effects of cocainism have nevertheless been observed in patients free from alcoholism or morphinism. According to Magnan and Saury, the chief bodily symptom in cocainism is the presence of certain cutaneous paræsthasiæ, such as sensations of worms, insects, or vermin on the skin or in wounds felt to be present in the skin, or in the feeling of foreign bodies (crystals, needles, &c.) as being beneath the skin. Next in frequency come hallucinations of sight, hearing, or smell, and finally mental confusion, with hypochondriacal and persecutory delusions. Some subjects, even after small doses, suffer from tetaniform seizures, hystero-epileptiform convulsions, and violent agitation or collapse. In one patient mentioned by MacFarlane as the subject of the cocaine habit, the delusion was that her skin was full of small insects and that her room was infested by serpents and reptiles.[70] While with morphia it is the enforced abstinence from the drug that produces an acute attack of mania or mental confusion or other form of collapse or exhaustion-psychosis, with cocaine, on the contrary, it is the prolonged use of the drug that develops the insanity, while abstinence gives rise to few noteworthy symptoms. The misuse of cocaine leads (says Peterson[71]) to the evolution of an acute hallucinatory paranoia.

There are a few other drugs the abuse of which may lead to characteristic mental troubles of varying degrees of gravity. Among these are ether, chloral, and cannabis indica. The insanity resulting from cannabis indica is extremely common in Egypt and other tropical countries. Thus Peterson records[72] that during a visit to the Cairo Asylum in the winter of 1891-92 he observed in the institution, with its population of 248 patients, 64 cases in which the insanity was due to the inhalation of haschish by smoking. "The symptoms produced were indigestion, diarrhœa, increased appetite, dilatation of the pupils, drooping eyelids, anæmia, general debility and delirium. The earliest mental symptom was marked and increasing timidity, sometimes amounting to *folie du doute*, or to agoraphobia."

Etherism resembles morphinism, but is a rarer disease and less grave. Ether-intoxication is accompanied by highly pleasurable hallucinations (Dieffenbach). According to Richet, hystero-epileptics are thrown into the third phase of their attacks (*attitudes passionelles*) by the inhalation of ether. The passion for ether (etheromania) is not marked by the same degree of irresistible craving for the drug, and its deprivation is not accompanied with the distress or the serious dangers and disturbances of function as in morphinism. Chloralism is, like morphinism, characterised by an irresistible tendency to take increasing doses, and by a genuine state of distress provoked by abstinence after its long-continued use, but the symptoms are less distressing and grave than in morphinism. Gastro-intestinal disorders are generally present, and there is some mental enfeeblement and stupidity. Sensorial and hallucinatory disturbances are, however, rare.

[67] The Chemistry of Common Life, vol. ii. [68] Degeneracy: page 118. [69] Ringer's Therapeutics, 1899. [70] Insomnia and its Therapeutics, 1890, page 176. [71] Peterson's Mental Diseases, Philadelphia, 1899. [72] New York Medical Record, May 21st, 1892.

